# Supplementary material for: Internal and external factors affecting vaccination coverage: Modeling the interactions between vaccine hesitancy, accessibility, and mandates
Source: PLOS Glob Public Health. 2023 Oct 4;3(10):e0001186. doi: 10.1371/journal.pgph.0001186 (PMC10550134; doi:10.1371/journal.pgph.0001186)
Supplement: S1 Text — (PDF) [file pgph.0001186.s001.pdf]

S1 Text: Detailed Methods

Here, we expand on the model proposed in [26]. We consider two cultural traits: a vaccination trait (**V**), and a vaccine attitude trait (**A**). Each individual can take one of two possible states for each trait, V^+ (vaccinated) or V^- (unvaccinated) and A^+ (vaccine confident) or A^- (vaccine hesitant), respectively. This results in four possible phenotypes: V^+A^+ (type 1: vaccinated and confident), V^+A^- (type 2: vaccinated and hesitant), V^-A^+ (type 3: unvaccinated and confident), and V^-A^- (type 4: unvaccinated and hesitant), whose frequencies in the population are denoted by x_1, x_2, x_3, and x_4, respectively, with $\sum_{i=1}^{4} x_i = 1$. (See **S1 Table** for subscript assignments).

The four phenotypes described produce sixteen possible mating pairs. The mating frequency, $m_{i,j}$ indicates the frequency of a mating between a parent of type i and the second parent of type j where $i, j = \{1, 2, 3, 4\}$ (**S1 Table**); for example, $m_{1,3}$ represents the mating frequency of V^+A^+ (x_1) and V^-A^+ (x_3). In this manuscript, we assume random mating, therefore individuals of different phenotypes mate with one another at a rate equal to the product of their frequencies.

Since the two traits (**A** and **V**) are transmitted vertically, for each phenotype we specify the probability that the mating produces an offspring of phenotype (V^+A^+). The vaccine confidence trait (A^+) is transmitted with probability C_n, and the vaccine hesitancy trait (A^-) is transmitted with probability $1-C_n$ (for $n = \{0, 1, 2, 3\}$ as shown in **S2 Table**). If $C_0 = 0$, two A^- parents will always produce A^- offspring, and if $C_3 = 1$, two A^+ parents will always produce A^+ offspring. However, if $C_0 > 0$, two A^- parents can produce A^+ offspring at some probability, and similarly if $C_3 < 1$, two A^+ parents can produce A^- offspring with some probability.

Transmission of vaccination (V^+ with probability $B_{m,n}$ for $m, n = \{0, 1, 2, 3\}$; **S2 Table**) is more complex, since parents' vaccine attitudes (**A**), in addition to their own vaccination states (**V**), can influence their behavior in vaccinating their offspring via a set of "influence parameters" that inform vaccination probabilities. The probability that each mating pair produces an offspring with the V^+ trait (i.e. vaccinates their offspring) is a scaled product of the influence of parental attitudes (c_n for $n = \{0, 1, 2, 3\}$) and the influence of parental vaccination states (b_m for $m = \{0, 1, 2, 3\}$) (**S2 Table**). For example, for mating pair $V^+A^+ \times V^+A^-$, their combined vaccination states ($V^+ \times V^+$) will influence vaccination behavior by b_3, and their combined attitude states, ($A^+ \times A^-$), will influence vaccination behavior by c_2. Therefore, a $V^+A^+ \times$

V^+A^- mating will produce a V^+ offspring with probability $B_{3,2} = c_2\left(\frac{1+b_3}{2}\right)$; this pair will also produce an A^+ offspring with probability C_2 based on their combined attitude states.

Transmission and influence probabilities are constant throughout a single simulation, with values ranging from 0 to 1. At baseline settings, the influence parameters b_m and c_n, and the transmission parameter C_n would take the values indicated in **Table 1**. In our model, vaccination probabilities are structured such that a couple's vaccine beliefs have a greater influence (c_n) on their likelihood of vaccinating their offspring than their own vaccination status (b_n). Therefore, offspring vaccination is guaranteed at some probability only if $c_n > 0$. We implement vaccine mandates and vaccine inaccessibility by modulating the influence of vaccine attitudes (c_n). We increase the influence parameter values of couples with at least one vaccine hesitant individual (c_0: $A^- \times A^-$, c_1: $A^- \times A^+$, c_2: $A^+ \times A^-$) to model a vaccine mandates or decreasing influence parameter values of couples with at least one vaccine confident individual (c_3: $A^+ \times A^+$, c_2: $A^+ \times A^-$, c_1: $A^- \times A^+$) to model vaccine inaccessibility. In other words, a vaccine mandate will make a vaccine-hesitant parent more likely to vaccinate their child, and vaccine inaccessibility will make a vaccine-confident parent less likely to vaccinate their child.

The cultural selection on vaccination is given by the parameter σ. After vertical cultural transmission has occurred, the frequency of the V^+A^+ and V^+A^- phenotypes are multiplied by $1+\sigma$. This parameter modulates whether there are more or fewer vaccinated individuals than expected: in other words, when $\sigma>0$, vaccinated individuals are more common in a set of offspring than would be expected strictly by parental beliefs and vaccination statuses. This cultural selection coefficient is structured to encompass both biological fitness and cultural selection pressures, including perceived risks or benefits of the vaccine itself, personal cost-benefit analyses of preventative health behaviors, and the structural or societal-level factors influencing vaccination rates [1,2]. Under the assumption that effects of herd immunity may lead to a reduction in vaccination behaviors—for example, the belief that vaccines are unnecessary when most others are vaccinated [3]—the cultural selection coefficient function in our model is vaccine-frequency-dependent. We calculate σ in each timestep as a function of the current vaccination coverage (frequency of V^+, i.e. $x_1 + x_2$), and in each simulation we specify σ_{max} as the maximum cultural selection pressure for getting vaccinated ($-1 \leq \sigma_{max} \leq 1$) (see the cultural selection coefficient function in **S1 Fig**). To incorporate this relationship into the model, we constructed a function by defining our assumptions (incorporating evolutionary game theory, e.g. the "free rider" problem) and then choosing curves with a trajectories that met pre-specified conditions: with unvaccinated individuals holding baseline fitness at 0, we assume

that when vaccination coverage is low, the real and perceived benefits of vaccination are highest, and thus, the cultural selection pressure is near σ_{max}, however, as vaccination coverage increases toward the level of herd immunity, the perceived benefits of vaccination decrease, represented as a reduction in the cultural selection pressure [4].

The model incorporates a second phase with oblique cultural transmission (i.e. influence from non-parental adults), in which individuals can change their inherited vaccine attitudes (**A**) due to influence from other adults in the population. There are two probabilities associated with attitude modulation: the probability that an vaccine hesitant (A^-) individual adopts the vaccine confident (A^+) state (A^- to A^+ transition probability, given by $A_{\rightarrow Confident}$ in **S2 Fig**), and the probability that an A^+ individual adopts the A^- state (A^+ to A^- transition probability, given by $A_{\rightarrow Hesitant}$ in **S2 Fig**). As with the strength of cultural selection (σ) described previously, the probability that offspring change their vaccine attitude is a function of the V^+ frequency in the population. As the frequency of vaccinated individuals (V^+) increases in the population, vaccine-confident individuals (A^+) are more likely to become hesitant ($A_{\rightarrow Hesitant}$ probability increases) and vaccine-hesitant individuals (A^-) are less likely to become confident ($A_{\rightarrow Confident}$ probability decreases). Similarly to the cultural selection function, the belief transition functions were generated by first choosing a function with a shape that aligned with our general assumptions and then modifying the function to fit specific criteria: 1) probabilities could approach zero, but not equal zero, 2) transition to supporting belief and transition to opposing belief are equally likely at 50% vaccination frequency, and, 3) that high vaccination frequencies (e.g. above herd-immunity levels of vaccination coverage) promote the transition to vaccine hesitancy [5,6]. The upper bound for the belief transition functions were set by calculating the percent difference between vaccine refusal rates in 1991 and 2004 in the United States to estimate transition probabilities between 1–2% [3]. By modulating the attitude transition probabilities according to the vaccination coverage in this manner, we assume that when vaccine coverage (V^+ frequency, $x_1 + x_2$) is low, disease occurrence is high and the negative effects of the disease are experienced widely, thus the benefits of being vaccinated (and the costs of not being vaccinated) are more evident [7,8]. As vaccination coverage (V^+) increases in the population, and thus disease occurrence is low, the benefits to being vaccinated are less obvious, while low-probability costs such as adverse reactions become more apparent and could be perceived as being riskier than the disease itself. Modulating both the attitude transition probabilities and the cultural selection coefficient according to the level of vaccination coverage in a population reflects that perceptions about the vaccine and its

associated effects on health could be meaningfully different in a population with high vaccination coverage than in one with low coverage.

To compute the frequency of a given phenotype in the next iteration, we sum the probability that each mating pair produces offspring of that phenotype over each of the sixteen possible mating pairs. Cultural selection (σ), described above, then operates on offspring with the V^+ trait. At the end of each timestep, the frequency of each phenotype is divided by the sum of all four frequencies, ensuring that the frequencies sum to 1. The full recursions, giving x_i' phenotype frequencies in the next iteration in terms of x_i in the current iteration, are given in **S2 Text**. If x_i' is equal to x_i, the system is at equilibrium. Unless otherwise stated, the model is initialized with phenotypic frequencies structured to represent those of the United States: x_1 (frequency of V^+A^+) = 0.81, x_2 (V^+A^-) = 0.1, x_3 (V^-A^+) = 0.07, x_4 (V^-A^-) = 0.02. These frequencies were estimated using reports of Measles-Mumps-Rubella (MMR) vaccination rates and estimates of vaccine attitude frequencies obtained from various sources in the literature [6,9] and the Centers of Disease Control ChildVax database [10,11].

Supplemental References

1. Pruitt RH, Kline PM, Kovaz RB. Perceived Barriers to Childhood Immunization Among Rural Populations. Journal of Community Health Nursing. 1995. pp. 65–72. doi:10.1207/s15327655jchn1202_1

2. Cavalli-Sforza LL, Feldman MW. Cultural transmission and evolution: a quantitative approach. Monogr Popul Biol. 1981;16: 1–388.

3. Omer SB, Salmon DA, Orenstein WA, deHart MP, Halsey N. Vaccine refusal, mandatory immunization, and the risks of vaccine-preventable diseases. N Engl J Med. 2009;360: 1981–1988.

4. Bauch CT, Bhattacharyya S. Evolutionary game theory and social learning can determine how vaccine scares unfold. PLoS Comput Biol. 2012;8: e1002452.

5. Jacobson RM, St Sauver JL, Finney Rutten LJ. Vaccine Hesitancy. Mayo Clin Proc. 2015;90: 1562–1568.

6. Kennedy AM, Brown CJ, Gust DA. Vaccine beliefs of parents who oppose compulsory vaccination. Public Health Rep. 2005;120: 252–258.

7. Gangarosa EJ, Galazka AM, Wolfe CR, Phillips LM, Gangarosa RE, Miller E, et al. Impact of anti-vaccine movements on pertussis control: the untold story. Lancet. 1998;351: 356–361.

8. Ozawa S, Mirelman A, Stack ML, Walker DG, Levine OS. Cost-effectiveness and economic benefits of vaccines in low- and middle-income countries: a systematic review. Vaccine. 2012;31: 96–108.

9. Leask J. Target the fence-sitters. Nature. 2011;473: 443–445.

10. Hill HA, Singleton JA, Yankey D, Elam-Evans LD, Cassandra Pingali S, Kang Y. Vaccination Coverage by Age 24 Months Among Children Born in 2015 and 2016 — National Immunization Survey-Child, United States, 2016–2018. MMWR. Morbidity and Mortality Weekly Report. 2019. pp. 913–918. doi:10.15585/mmwr.mm6841e2

11. Hill HA, Elam-Evans LD, Yankey D, Singleton JA, Kang Y. Vaccination Coverage Among Children Aged 19–35 Months — United States, 2016. MMWR. Morbidity and Mortality Weekly Report. 2017. pp. 1171–1177. doi:10.15585/mmwr.mm6643a3
