# Supplementary material for: Internal and external factors affecting vaccination coverage: Modeling the interactions between vaccine hesitancy, accessibility, and mandates
Source: PLOS Glob Public Health. 2023 Oct 4;3(10):e0001186. doi: 10.1371/journal.pgph.0001186 (PMC10550134; doi:10.1371/journal.pgph.0001186)
Supplement: S2 Text — (PDF) [file pgph.0001186.s002.pdf]

S2 Text: Recursions for Vaccine Niche Construction

$$\bar{w}x_1' = (1+\sigma_1)\big(m_{11}B_{3,3}C_3 + m_{12}B_{3,2}C_2 + m_{21}B_{3,1}C_1 + m_{13}B_{2,3}C_3 + m_{31}B_{1,3}C_3 + m_{14}B_{2,2}C_2 + m_{41}B_{1,1}C_1 + m_{22}B_{3,0}C_0 + m_{23}B_{2,1}C_1 + m_{32}B_{1,2}C_2 + m_{24}B_{2,0}C_0 + m_{42}B_{1,0}C_0 + m_{33}B_{0,3}C_3 + m_{34}B_{0,2}C_2 + m_{43}B_{0,1}C_1 + m_{44}B_{0,0}C_0\big)$$

$$\bar{w}x_2' = (1+\sigma_1)\big(m_{11}B_{3,3}(1-C_3) + m_{12}B_{3,2}(1-C_2) + m_{21}B_{3,1}(1-C_1) + m_{13}B_{2,3}(1-C_3) + m_{31}B_{1,3}(1-C_3) + m_{14}B_{2,2}(1-C_2) + m_{41}B_{1,1}(1-C_1) + m_{22}B_{3,0}(1-C_0) + m_{23}B_{2,1}(1-C_1) + m_{32}B_{1,2}(1-C_2) + m_{24}B_{2,0}(1-C_0) + m_{42}B_{1,0}(1-C_0) + m_{33}B_{0,3}(1-C_3) + m_{34}B_{0,2}(1-C_2) + m_{43}B_{0,1}(1-C_1) + m_{44}B_{0,0}(1-C_0)\big)$$

$$\bar{w}x_3' = \big(m_{11}(1-B_{3,3})C_3 + m_{12}(1-B_{3,2})C_2 + m_{21}(1-B_{3,1})C_1 + m_{13}(1-B_{2,3})C_3 + m_{31}(1-B_{1,3})C_3 + m_{14}(1-B_{2,2})C_2 + m_{41}(1-B_{1,1})C_1 + m_{22}(1-B_{3,0})C_0 + m_{23}(1-B_{2,1})C_1 + m_{32}(1-B_{1,2})C_2 + m_{24}(1-B_{2,0})C_0 + m_{42}(1-B_{1,0})C_0 + m_{33}(1-B_{0,3})C_3 + m_{34}(1-B_{0,2})C_2 + m_{43}(1-B_{0,1})C_1 + m_{44}(1-B_{0,0})C_0\big)$$

$$\bar{w}x_4' = \big(m_{11}(1-B_{3,3})(1-C_3) + m_{12}(1-B_{3,2})(1-C_2) + m_{21}(1-B_{3,1})(1-C_1) + m_{13}(1-B_{2,3})(1-C_3) + m_{31}(1-B_{1,3})(1-C_3) + m_{14}(1-B_{2,2})(1-C_2) + m_{41}(1-B_{1,1})(1-C_1) + m_{22}(1-B_{3,0})(1-C_0) + m_{23}(1-B_{2,1})(1-C_1) + m_{32}(1-B_{1,2})(1-C_2) + m_{24}(1-B_{2,0})(1-C_0) + m_{42}(1-B_{1,0})(1-C_0) + m_{33}(1-B_{0,3})(1-C_3) + m_{34}(1-B_{0,2})(1-C_2) + m_{43}(1-B_{0,1})(1-C_1) + m_{44}(1-B_{0,0})(1-C_0)\big)$$
