# Supplementary figures and images for: Internal and external factors affecting vaccination coverage: Modeling the interactions between vaccine hesitancy, accessibility, and mandates
Source: PLOS Glob Public Health. 2023 Oct 4;3(10):e0001186. doi: 10.1371/journal.pgph.0001186 (PMC10550134; doi:10.1371/journal.pgph.0001186)

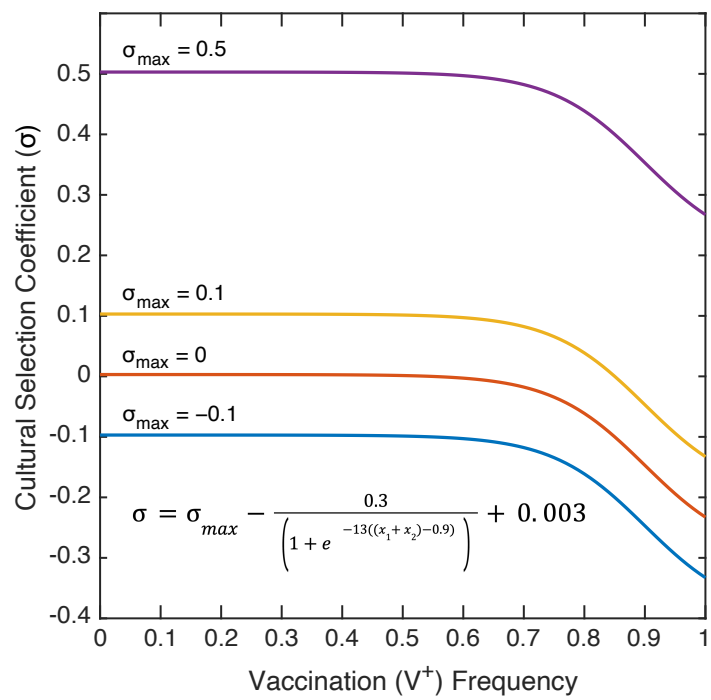

Supplement: S1 Fig — The cultural selection coefficient considers both health and non-health related effects, and the function was constructed by fitting a curve to specified conditions. The selection coefficient (σ; vertical-axis) is dependent on the frequency of vaccinated individuals (V+) in the population (horizontal-axis). σmax is the maximum cultural selection coefficient associated with being vaccinated. Perceived vaccine benefit is reduced as vaccination coverage increases, since the negative effects of the disease will be less apparent. (PDF) [file pgph.0001186.s003.pdf]

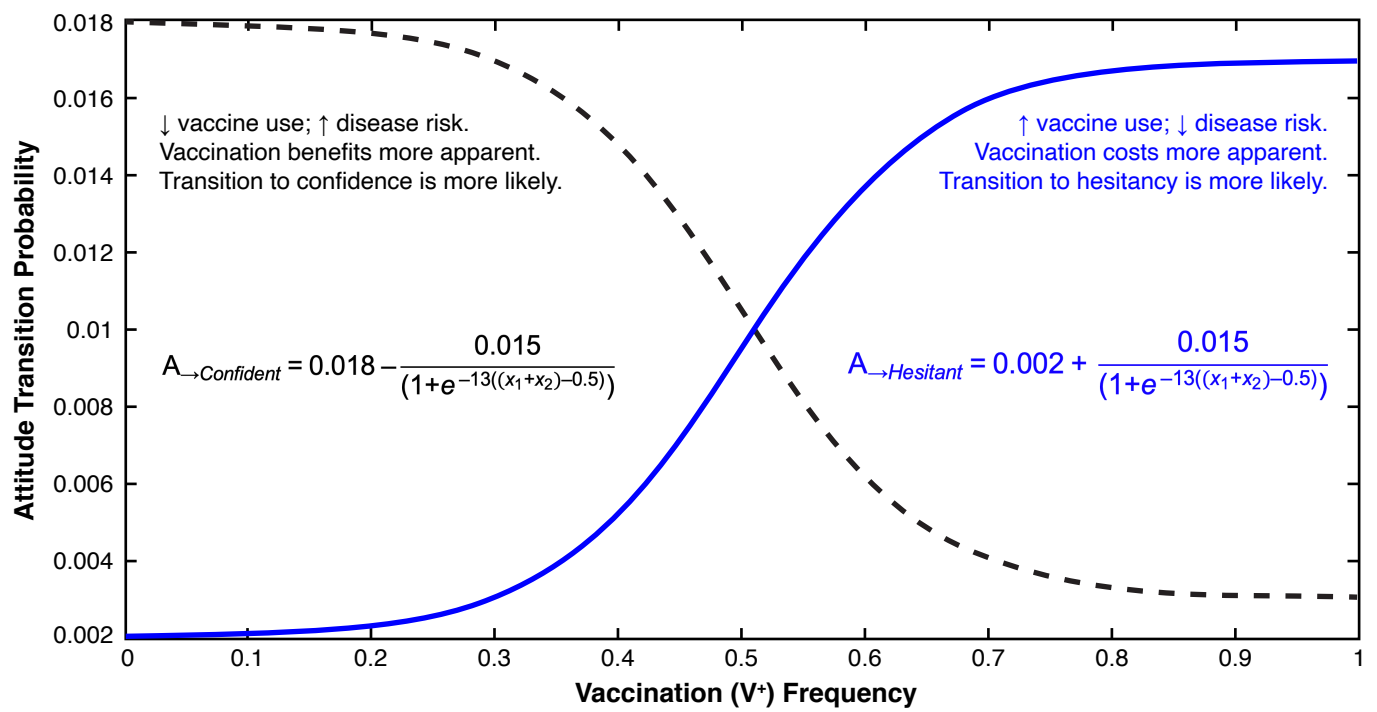

Supplement: S2 Fig — Attitude transition probability functions were constructed by fitting a curve to specified values. Attitude transition probability (vertical axis) is a function of the vaccination frequency in the population (V+; horizontal axis). The probability that a vaccine hesitant individual adopts vaccine confidence (A− to A+ transition probability, shown in dashed black) is determined by the function A→Confident, and the probability that a vaccine confident individual adopts vaccine hesitancy (A+ to A− transition probability, shown with a solid blue line) is determined by the function A→Hesitant. (PDF) [file pgph.0001186.s004.pdf]

**A****Functions**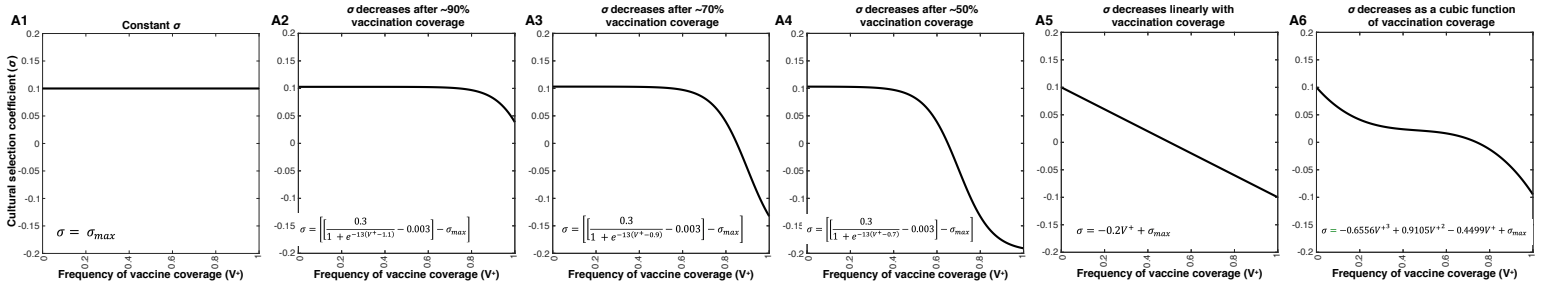**B****No mandate**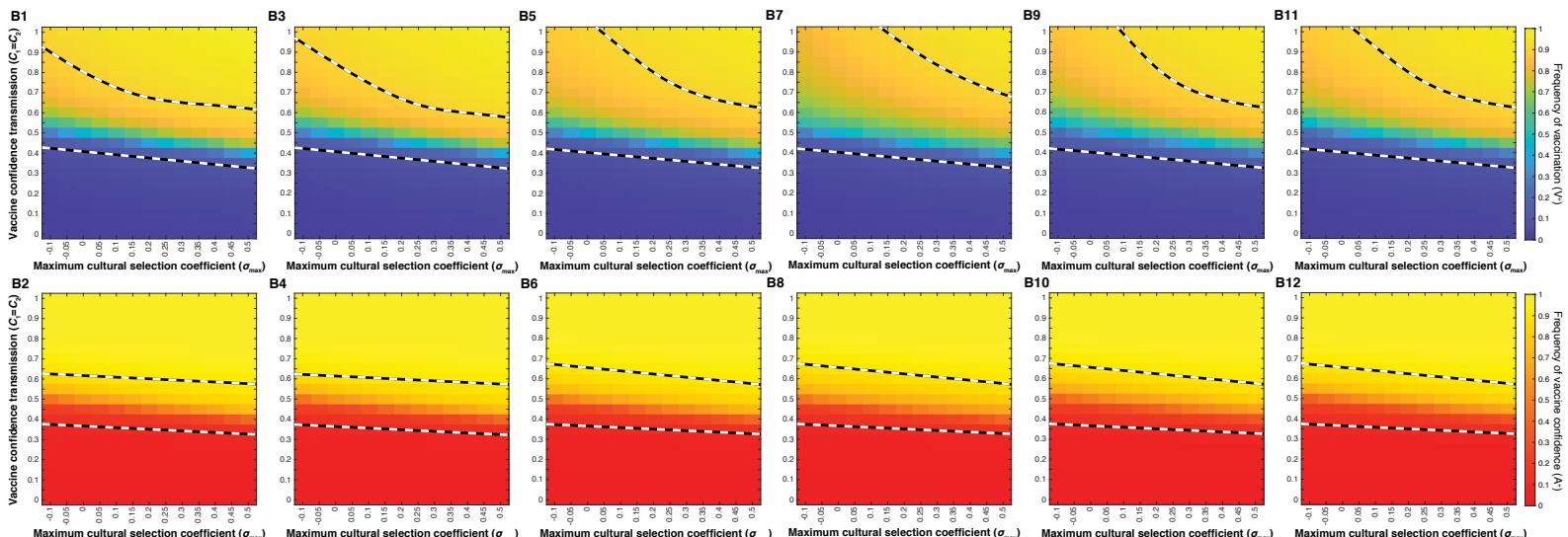**C****Less strict mandate**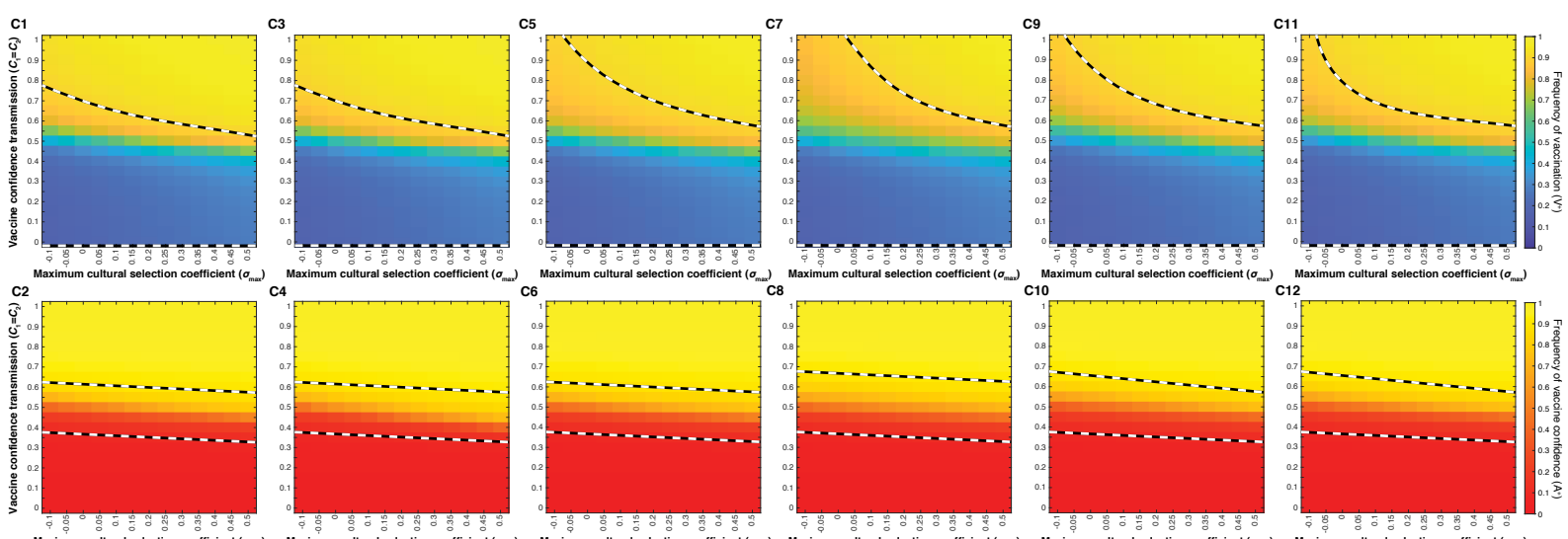**D****Inaccessible vaccine**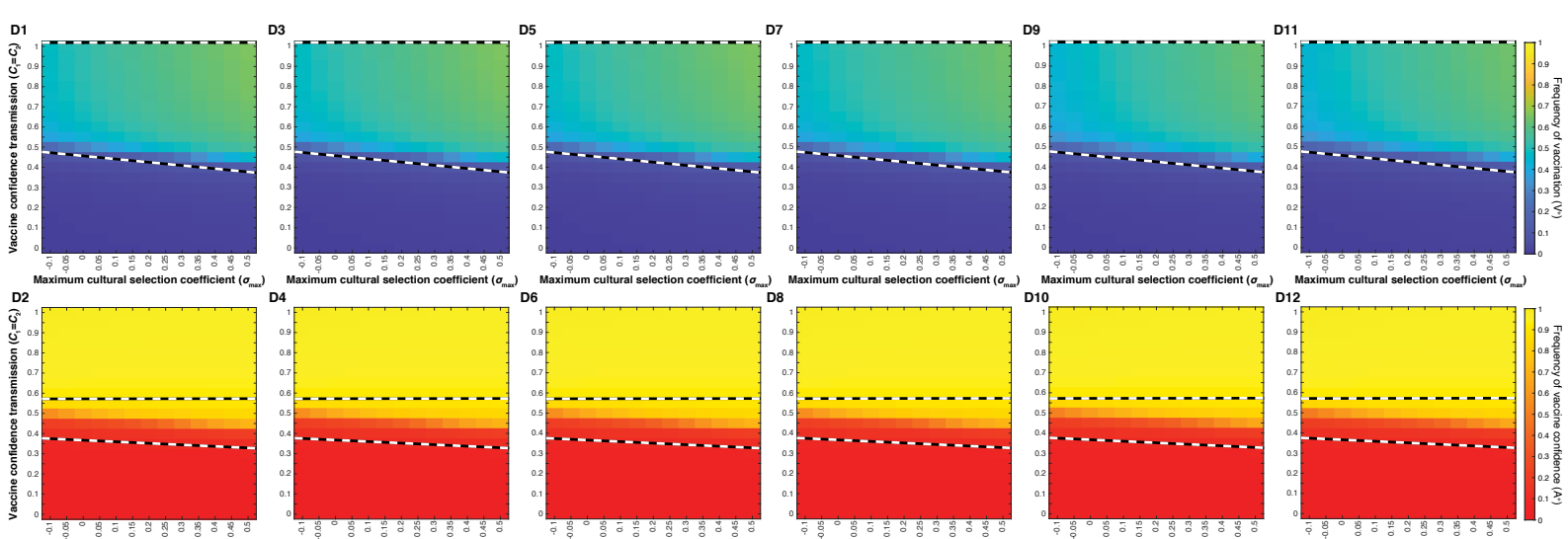

Supplement: S3 Fig — Heatmaps showing equilibrium vaccine coverage and vaccine confidence levels with an accessible vaccine and no mandate (Section B), with an accessible vaccine and a less strict mandate (Section C) and an environment with vaccines somewhat inaccessible (Section D), employing various cultural selection (σ) functions: (A1) σ does not depend on vaccination coverage, (A2) σ decreases after a high herd-immunity threshold of ~90% coverage, (A3) σ decreases after a medium herd-immunity threshold of ~70% coverage (baseline function), (A4) σ decreases after a low herd-immunity threshold of ~50% coverage, (A5) σ decreases linearly as vaccination coverage increases, (A6) σ decreases according to a cubic function. We vary C1 = C2 (confidence transmission probability of mixed-attitude couples) on the vertical axis, and maximum selection coefficient σmax (indicative of the perceived value of vaccinating offspring) on the horizontal axis. Unspecified parameters are given in Table 1 with σmax held at 0.1 for all functions shown in Section A but varied in the heatmaps in Sections B-D. Black and white dashed lines indicate the area of the heat maps in which vaccination and confidence frequencies equilibrate between 0.1 and 0.9. (PDF) [file pgph.0001186.s005.pdf]
