# Supplementary material for: Internal and external factors affecting vaccination coverage: Modeling the interactions between vaccine hesitancy, accessibility, and mandates
Source: PLOS Glob Public Health. 2023 Oct 4;3(10):e0001186. doi: 10.1371/journal.pgph.0001186 (PMC10550134; doi:10.1371/journal.pgph.0001186)
Supplement: S1 Table — Demonstrating the trait presence (+) and absence (–) combinations associated with m, n subscripts. For example, the + ×–combinations is associated with m and n subscript value 2: an A+ × A−pairing transmits A+ at probability C2. This rule applies to parameters Cn, bm, Bm,n, cn, as shown in S2 Table. (PDF) [file pgph.0001186.s006.pdf]

S1 Table: Presence (+) and absence (–) subscript assignments. Demonstrating the trait presence (+) and absence (–) combinations associated with m, n subscripts. For example, the + × – combinations is associated with m and n subscript value 2: an $A^+ \times A^-$ pairing transmits A^+ at probability C_2. This rule applies to parameters C_n, b_m, $B_{m,n}$, c_n, as shown in **S2 Table**.

Subscript Value (*m*, *n*; e.g. b_m, C_n)	Associated Pairing (e.g. V × V, A × A)
0	– × –
1	– × +
2	+ × –
3	+ × +
