# Supplementary material for: Internal and external factors affecting vaccination coverage: Modeling the interactions between vaccine hesitancy, accessibility, and mandates
Source: PLOS Glob Public Health. 2023 Oct 4;3(10):e0001186. doi: 10.1371/journal.pgph.0001186 (PMC10550134; doi:10.1371/journal.pgph.0001186)
Supplement: S2 Table — For each mating, we give the probability of transmitting each trait, and corresponding influence parameters. The probability of vaccinating an offspring, Bm,n, depends on both the parents’ vaccination state (V+: vaccinated; V−: unvaccinated) and their attitude state (A+: vaccine confident; A−: vaccine hesitant). Bm,n is informed by the influence of parents’ vaccination states (V) on their decision to vaccinate (bm) and by the influence of their vaccine attitudes (A) on their decision to vaccinate (cn). For each parental pairing, the probability of not vaccinating an offspring is 1 –Bm,n. Each pairing transmits confidence in vaccines at a rate Cn, and hesitancy at rate 1 –Cn. The parameters bm, cn, and Cn are set as constants for each simulation, and Bm,n is calculated from these. (PDF) [file pgph.0001186.s007.pdf]

S2 Table: Probabilities of trait transmission to offspring from cultural trait pairings.

For each mating, we give the probability of transmitting each trait, and corresponding influence parameters. The probability of vaccinating an offspring, $B_{m,n}$, depends on both the parents' vaccination state (V^+: vaccinated; V^-: unvaccinated) and their attitude state (A^+: vaccine confident; A^-: vaccine hesitant). $B_{m,n}$ is informed by the influence of parents' vaccination states (**V**) on their decision to vaccinate (b_m) and by the influence of their vaccine attitudes (**A**) on their decision to vaccinate (c_n). For each parental pairing, the probability of not vaccinating an offspring is $1 - B_{m,n}$. Each pairing transmits confidence in vaccines at a rate C_n, and hesitancy at rate $1 - C_n$. The parameters b_m, c_n, and C_n are set as constants for each simulation, and $B_{m,n}$ is calculated from these.

	**Trait Transmission Probabilities**				**Influence of parental vaccination and attitudes on offspring vaccination**	
**Mating pair**	**Offspring vaccination (V^+) probability**	**V^- offspring probability**	**A^+ offspring probability**	**A^- offspring probability**	**V influence (*m*)**	**A influence (*n*)**
$V^+A^+ \times V^+A^+$	$B_{m=3,n=3} = c_3\left(\frac{1+b_3}{2}\right)$	$1 - B_{3,3}$	C_3	$1 - C_3$	b_3	c_3
$V^+A^+ \times V^+A^-$	$B_{3,2} = c_2\left(\frac{1+b_3}{2}\right)$	$1 - B_{3,2}$	C_2	$1 - C_2$	b_3	c_2
$V^+A^- \times V^+A^+$	$B_{3,1} = c_1\left(\frac{1+b_3}{2}\right)$	$1 - B_{3,1}$	C_1	$1 - C_1$	b_3	c_1
$V^+A^- \times V^+A^-$	$B_{3,0} = c_0\left(\frac{1+b_3}{2}\right)$	$1 - B_{3,0}$	C_0	$1 - C_0$	b_3	c_0
$V^+A^+ \times V^-A^+$	$B_{2,3} = c_3\left(\frac{1+b_2}{2}\right)$	$1 - B_{2,3}$	C_3	$1 - C_3$	b_2	c_3
$V^+A^+ \times V^-A^-$	$B_{2,2} = c_2\left(\frac{1+b_2}{2}\right)$	$1 - B_{2,2}$	C_2	$1 - C_2$	b_2	c_2
$V^+A^- \times V^-A^+$	$B_{2,1} = c_1\left(\frac{1+b_2}{2}\right)$	$1 - B_{2,1}$	C_1	$1 - C_1$	b_2	c_1
$V^+A^- \times V^-A^-$	$B_{2,0} = c_0\left(\frac{1+b_2}{2}\right)$	$1 - B_{2,0}$	C_0	$1 - C_0$	b_2	c_0
$V^-A^+ \times V^+A^+$	$B_{1,3} = c_3\left(\frac{1+b_1}{2}\right)$	$1 - B_{1,3}$	C_3	$1 - C_3$	b_1	c_3

$V^-A^+ \times V^+A^-$	$B_{1,2} = c_2\left(\frac{1+b_1}{2}\right)$	$1 - B_{1,2}$	C_2	$1 - C_2$	b_1	c_2
$V^-A^- \times V^+A^+$	$B_{1,1} = c_1\left(\frac{1+b_1}{2}\right)$	$1 - B_{1,1}$	C_1	$1 - C_1$	b_1	c_1
$V^-A^- \times V^+A^-$	$B_{1,0} = c_0\left(\frac{1+b_1}{2}\right)$	$1 - B_{1,0}$	C_0	$1 - C_0$	b_1	c_0
$V^-A^+ \times V^-A^+$	$B_{0,3} = c_3\left(\frac{1+b_0}{2}\right)$	$1 - B_{0,3}$	C_3	$1 - C_3$	b_0	c_3
$V^-A^+ \times V^-A^-$	$B_{0,2} = c_2\left(\frac{1+b_0}{2}\right)$	$1 - B_{0,2}$	C_2	$1 - C_2$	b_0	c_2
$V^-A^- \times V^-A^+$	$B_{0,1} = c_1\left(\frac{1+b_0}{2}\right)$	$1 - B_{0,1}$	C_1	$1 - C_1$	b_0	c_1
$V^-A^- \times V^-A^-$	$B_{0,0} = c_0\left(\frac{1+b_0}{2}\right)$	$1 - B_{0,0}$	C_0	$1 - C_0$	b_0	c_0
