# Supplementary material for: Internal and external factors affecting vaccination coverage: Modeling the interactions between vaccine hesitancy, accessibility, and mandates
Source: PLOS Glob Public Health. 2023 Oct 4;3(10):e0001186. doi: 10.1371/journal.pgph.0001186 (PMC10550134; doi:10.1371/journal.pgph.0001186)
Supplement: S3 Table — Each probability was grouped according to baseline vaccination probability calculations. All probabilities in a group hold the value assigned to that group in the range, as shown. Cn probabilities were assigned values as shown, with C0 taking the lowest value in the range and C3 taking the highest. The lowest probability range group is given as an example of value assignment. (PDF) [file pgph.0001186.s008.pdf]

S3 Table: Probability range shift assignments

Each probability was grouped according to baseline vaccination probability calculations. All probabilities in a group hold the value assigned to that group in the range, as shown. C_n probabilities were assigned values as shown, with C_0 taking the lowest value in the range and C_3 taking the highest. The lowest probability range group is given as an example of value assignment.

	Range: Low → High			
Parameters	C_0	C_1	C_2	C_3
Example value (range 0.1–0.4)	0.1	0.2	0.3	0.4
