# Supplementary material for: Internal and external factors affecting vaccination coverage: Modeling the interactions between vaccine hesitancy, accessibility, and mandates
Source: PLOS Glob Public Health. 2023 Oct 4;3(10):e0001186. doi: 10.1371/journal.pgph.0001186 (PMC10550134; doi:10.1371/journal.pgph.0001186)
Supplement: S4 Table — The mean and median of vaccination coverage and vaccine confidence levels at equilibrium were calculated for the section of the heatmaps in Fig 2 for which C1 = C2 < 0.5 (blue in vaccination coverage heatmaps; red in the confidence level heatmaps). (PDF) [file pgph.0001186.s009.pdf]

S4 Table: Quantitative differences between equilibrium frequencies with low transmission of vaccine confidence

The mean and median of vaccination coverage and vaccine confidence levels at equilibrium were calculated for the section of the heatmaps in **Fig 2** for which $C_1 = C_2 < 0.5$ (blue in vaccination coverage heatmaps; red in the confidence level heatmaps).

Vaccination Coverage below $C_1 = C_2 < 0.5$	**No Mandate**	**Less Strict Mandate**	**Vaccine Inaccessible**
**Mean**	9.031%	27.723%	5.032%
**Median**	4.047%	25.872%	2.578%
Confidence Levels below $C_1 = C_2 < 0.5$			
**Mean**	10.875%	9.092%	9.927%
**Median**	5.262%	5.115%	5.178%
